# Post Zygotic, Somatic, Deletion in KERATIN 1 V1 Domain Generates Structural Alteration of the K1/K10 Dimer, Producing a Monolateral Palmar Epidermolytic Nevus

**DOI:** 10.3390/ijms22136901

**Published:** 2021-06-27

**Authors:** Sabrina Caporali, Biagio Didona, Mauro Paradisi, Alessandro Mauriello, Elena Campione, Mattia Falconi, Federico Iacovelli, Marilena Minieri, Massimo Pieri, Sergio Bernardini, Alessandro Terrinoni

**Affiliations:** 1 Department of Industrial Engineering, University of Rome Tor Vergata, 00133 Rome, Italy; sabrina.caporali93@gmail.com; 2 Fondazione Luigi Maria Monti, IDI-IRCCS, 00167 Rome, Italy; b.didona@idi.it; 3 Department of Experimental Medicine, University of Tor Vergata, 00133 Rome, Italy; aparad78@gmail.com (M.P.); alessandro.mauriello@uniroma2.it (A.M.); marilenami@gmail.com (M.M.); massimo.pieri@uniroma2.it (M.P.); bernardini@med.uniroma2.it (S.B.); 4 Department of Systems Medicine, University of Tor Vergata, 00133 Rome, Italy; campioneelena@hotmail.com; 5 Department of Biology, University of Rome Tor Vergata, Via della Ricerca Scientifica, 00133 Rome, Italy; falconi@uniroma2.it (M.F.); federico.iacovelli@uniroma2.it (F.I.)

**Keywords:** EPPK, NEPPK, keratins, genodermatosis, mosaic, keratin structure, KIF

## Abstract

Palmoplantar keratodermas (PPKs) are characterized by thickness of stratum corneum and epidermal hyperkeratosis localized in palms and soles. PPKs can be epidermolytic (EPPK) or non epidermolytic (NEPPK). Specific mutations of keratin 16 (K16) and keratin 1 (K1) have been associated to EPPK, and NEPPK. Cases of mosaicism in PPKs due to somatic keratin mutations have also been described in scientific literature. We evaluated a patient presenting hyperkeratosis localized monolaterally in the right palmar area, characterized by linear yellowish hyperkeratotic lesions following the Blaschko lines. No other relatives of the patient showed any dermatological disease. Light and confocal histological analysis confirmed the presence of epidermolityic hyperkeratosis. Genetic analysis performed demonstrates the heterozygous deletion NM_006121.4:r.274_472del for a total of 198 nucleotides, in *KRT1* cDNA obtained by a palmar lesional skin biopsy, corresponding to the protein mutation NP_006112.3:p.Gly71_Gly137del. DNA extracted from peripheral blood lymphocytes did not display the presence of the mutation. These results suggest a somatic mutation causing an alteration in K1 N-terminal variable domain (V1). The deleted sequence involves the ISIS subdomain, containing a lysine residue already described as fundamental for epidermal transglutaminases in the crosslinking of IF cytoskeleton. Moreover, a computational analysis of the wild-type and V1-mutated K1/K10 keratin dimers, suggests an unusual interaction between these keratin filaments. The mutation taster in silico analysis also returned a high probability for a deleterious mutation. These data demonstrate once again the importance of the head domain (V1) of K1 in the formation of a functional keratinocyte cytoskeleton. Moreover, this is a further demonstration of the presence of somatic mutations arising in later stages of the embryogenesis, generating a mosaic phenotype.

## 1. Introduction

Molecular genetic defects of several inherited skin diseases (Genodermatosis) have been characterized in the last decades [1,2]. Several mutations have been found in genes coding for structural epidermal protein keratins, involved in epidermal differentiation and maintenance. These genetic defects often lead to incorrect interaction among the basic components of the intermediate filament (IF) network, generating an incomplete or abnormal cytoskeleton. The resulting phenotype is generally characterized by the type, the localization and the role of the protein involved. Bullous congenital ichthyosiform erythroderma (BCIE, OMIM 113800), also known as epidermolytic hyperkeratosis (EH) and now classified inside the class of Keratinopatic Ichthyoses [3], is an autosomal dominant disorder of the skin associated to mutations in *KRT1* and *KRT10* genes. BCIE is characterized by the presence of erythroderma and skin blistering at birth, later replaced by a diffuse hyperkeratosis of the skin and, by the presence of palmoplantar keratoderma (PPK).

PPKs are clinically characterized by the thickening and hyperkeratosis of palm and sole epidermis [4]. Different types of PPKs have been described, basing on the presence of other extracutaneous manifestations. Syndromic PPKs are characterized by the involvement of nails, teeth and other organs, while non-syndromic PPKs affect only the palmoplantar region. PPKs can be distinguished in NEPPK (Non-epidermolytic palmoplantar keratoderma) or EPPK (epidermolytic palmoplantar keratoderma). Both NEPPK and EPPK show, respectively, the absence or the presence of epidermolysis. Thickness of stratum corneum and epidermal hyperplasia of palms and soles often appear early after birth. Non syndromic PPKs are mainly caused by keratin defects. From literature, mutations in both *KRT1* that *KRT9* have been found in EPKK [5,6], while K1 mutations have been also reported in NEPPK [7,8]. Another differentiation-specific keratin, K16, plays a role in PPKs, the defects of which are associated to focal non-epidermolytic palmoplantar keratoderma (FNEPPK) [9].

Keratins are major structural proteins forming intermediate filaments (KIFs) of the functional keratinocyte cytoskeleton. Keratins are classified in keratin type I or acidic (K9-K20) and keratin type II or basic (K1-K8), which form heterodimer complexes. Structurally, they contain a central coiled-coil rod domain with four alpha-helical segments (1A, 1B, 2A, and 2B) separated by three non-helical linker elements (L1, L12, and L2) [10]. These domains are highly conserved among keratins of the same class and among species. The rod domain starts and ends with two short, highly conserved amino acid sequences, known as helix initiation peptide (HIP) and helix termination peptide (HTP), [11,12,13]. The rod domain contains a repetition of an amino acid heptad sequence (a-b-c-d-e-f-g). Positions “a” and “d” give rise to hydrophobic interactions, and positions “e” and “g” to ionic hydrogen interactions that highly stabilize the structure. “a” residues, through hydrophobic interactions, interact with amino acids located in the “d” position of the heterodimer partner molecule, stabilizing the two-chain coiled-coil molecules. In the K1/K10 pair, amino acid changes, modifying the initial coupling of keratin dimers [14,15,16], generate molecular distortion in the [11] alpha-helical structure, with deleterious effects on KIF formation and on the integrity of the epidermis [17,18]. For this reason, keratin mutations associated to skin diseases are frequently mapped to the HIP and in the HTP hot spots [6,19]. In contrast to the rod domain, the two flanking non-helical head (V1) and tail (V2) domains, varies in length between different keratins, for example V1 domain is composed of 180 residues in K1 and 80 in K18. The V1 domain of epithelial keratins also show a high variability in sequence, they typically contain high numbers of glycine and few cysteine residues, but maintain in common the presence of a glycine loop structure. Importantly, in keratins such as K1, the head domain can be further divided into three different subdomains named E1 (N-terminal), V1 (central) and H1 that C-terminally flanks the V1 domain and N-terminally the central rod domain [16].

Even if mutations are frequently located in the 1A and 2B domains, mutations compromising the correct folding of total C-terminal 2B/V2 hetero domain of keratin type I/II couple can led to tonofilament aggregation [20] and cytoskeleton instability. Indeed, also alterations in V1 domain have been demonstrated to be important for filament integrity [21]. Specifically, alteration in V1 domain of K1 has been demonstrated to give rise also to BCIE. In this case, the mutation affects a lysine residue in a functional subdomain containing the sequence named ISIS-box [7]. This conserved region lysine-rich is important for transglutaminase, transglutaminase crosslink through isopeptide-bonded keratin intermediate filaments, thus generating cytoskeleton integrity [22]. This evidence further suggests the important role of keratin head and tail domains.

Interestingly, the investigation of skin diseases due to keratin mutation offer the possibility to study genetic mosaics. The cutaneous mosaicism offers a unique opportunity to investigate this genetic disorder, since the lesional skin can be easily identified and the proband genes can be analyzed from a skin biopsy. The predictable embryonic cell migration patterns of ectodermal progenitors, leading to the formation of the cutaneous ectoderm, should theoretically result in linear bands known as the lines of Blaschko as the setting of embryonic somatic mutation [23,24]. However, a new classification system of mosaic skin disorders based on genomic versus epigenetic etiology, and segmental versus nonsegmental distribution, was proposed [25].

In several cutaneous mosaics, the lesions have a segmental presentation; this reflects the migration pattern of epidermal progenitors during embryogenesis. Most of them resemble the lines of Blaschko and respect the midline, while others occur in non-blaschkoid but also distinguishable segmental patterns. Indeed, other cutaneous mosaic disorders demonstrate no predictable pattern [25].

In this study, we analyzed a female patient affected by non-syndromic palmoplantar keratoderma, localized mono-laterally in the right palmar region. This study will be useful to define the genetic alterations leading to skin diseases that cannot be associated to blaschkoid or the segmental mosaic.

## 2. Results

### 2.1. Patient Presentation

**Figure 1:**
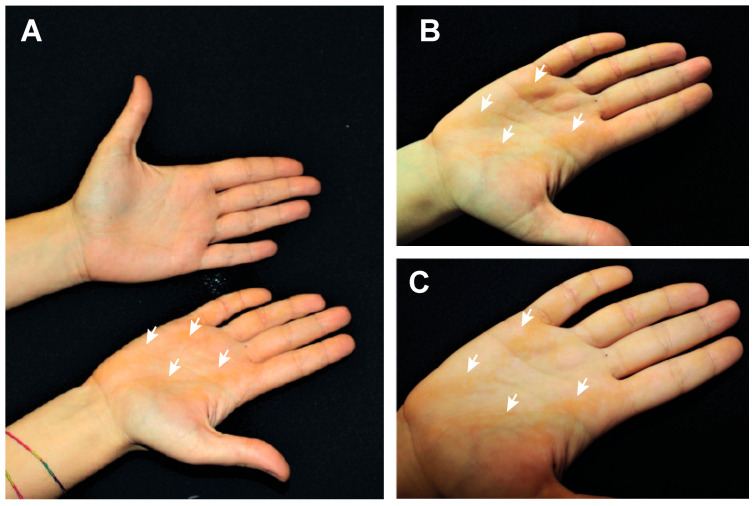
Patient presentation. (**A**), picture of both the patient’s hands showing hyperkeratotic lesions only in the right palm. (**B**,**C**) pictures of patient’s right palm showing the hyperkeratotic lesions (white arrows), with a nevoid distribution.

We analyzed a 21-year-old female patient showing linear verrucous lesions only in the right palmar area (Figure 1A). Consanguinity of parents was evaluated and excluded. No other relatives (father, mother and sister) are affected by any dermatological condition. The patient had lesions that appeared since childhood. In our clinical examination, multiple, well demarcated, hyperkeratotic and yellowish streaks were evident in the right palm, following the Blaschko lines, but completely absent in the left palm (Figure 1A). No similar lesions were observed in other cutaneous regions. Moreover, a complete screening of all skin district including flexures did not identify hyperkeratosis or ichthyotic lesions. Interestingly, the lesions have a nevoid distribution that intercross the palmar creases. They consist of three main stripes localizing across the radial longitudinal crease, just below the thenar eminence, another localized along the middle and thenar creases, and one more external from the hypothenar eminence to the distal transverse crease.

After this first examination, a further patient reevaluation was conducted to check the possible presence of leukoplakia and of signs of laryngeal squamous cell carcinoma (LSCC). This is because the molecular analysis showed the presence of a single nucleotide polymorphism (SNP) identified by rs14024 recently associated to an increased risk of LSCC [26]. The dermatological and otolaryngological screening did not reveal abnormalities.

### 2.2. Histological Analysis

#### 2.2.1. Light Microscopy

**Figure 2:**
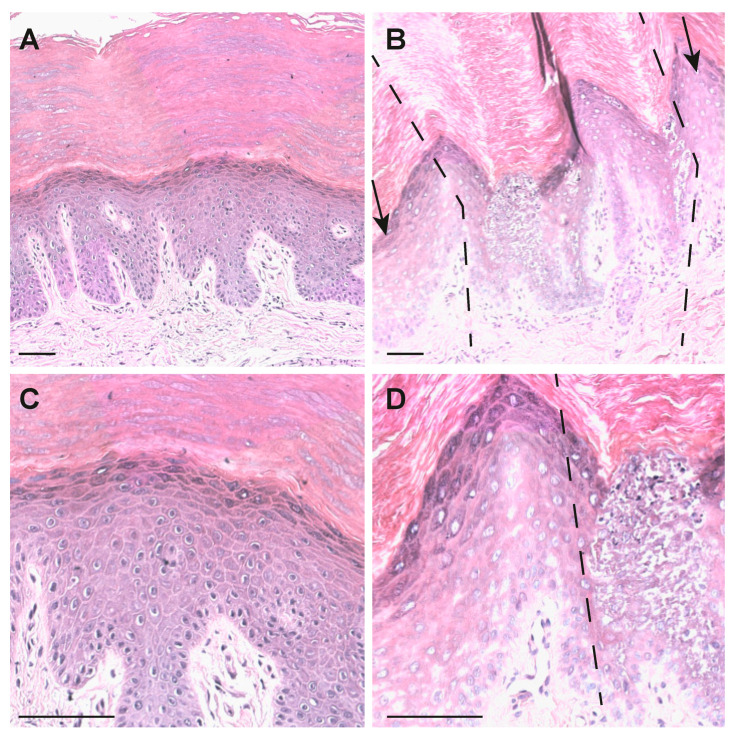
HE staining of palmar skin. (**A**) Control palmar skin. (**B**) Patient palmar skin. Dashed lines and arrows indicate the joint between nevoid lesion and non or low lesional skin (10× objective). (**C**) Control palmar skin. (**D**) Patient palmar skin (20× objective), low lesional skin is on the left side of the dotted line. Bars = 50 μm.

A skin biopsy of the patient was taken to analyze the structure of hyperkeratotic epidermis. HE staining showed a well-defined band of hyperkeratosis (Figure 2B,D), vacuolar degeneration of keratinocytes of the spinosum and granular layers; pyknotic nuclei, an increase number of keratohyaline granuli, was also seen compared with the normal control (Figure 2A,C). The differences, compared with adjacent not-involved skin (Figure 2B,D, dashed lines) and normal control skin, are evident. In fact, patient sections showed a thicker stratum corneum (Figure 2B) compared with a normal control (Figure 2A). Moreover, a lower degree of interconnection of the component in this district is evident. No parakeratosis is observed. An evident epidermolysis is present in involved areas of patient skin (Figure 2B,C) involving both suprabasal spinous and granular layers. Detachment of part of the corneum layer was also observed from this palmar biopsy, as well as a high degree of acanthosis and epidermolytic hyperkeratosis.

From the histological findings, the diagnosis is compatible with an epidermolytic form of palmoplantar keratoderma with a possible keratin involvement.

#### 2.2.2. Confocal Microscopy

**Figure 3:**
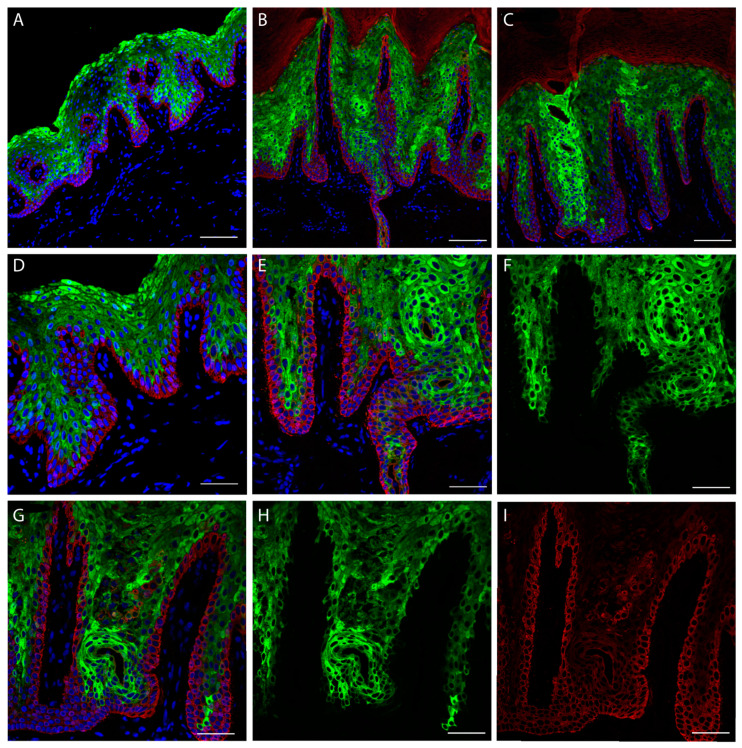
Skin confocal immunofluorescence analysis. Staining was performed using antibodies against K14 (red) and K1 (green). (**A**) Control palmar skin. (**B**,**C**) Patient palmar skin (20× objective). (**D**) Normal palmar skin (40× objective). (**E**), Patient palmar skin with K1 and K14 staining merged with DAPI (40× objective, also for (**F**–**I)**. (**F**) Capture only showing K1 distribution. (**G**) Patient skin with merged channels showing K14, K1 and DAPI. (**H**) Patient skin showing only K1 staining. (**I**) Patient skin showing only K14 staining. (**A**–**F**) Scale bar = 100 µm. (**G**–**I**) Scale bar = 50 µm.

The ultrastructural analysis of patient skin was performed using a confocal microscope. Fluorescent antibodies directed against cytoskeletal proteins was used to detect epidermal structure. K1 staining (Figure 3, Green) shows positivity for an elevated number of epidermal layers in patient sections (Figure 3B,C) compared with the control (Figure 3A), leading to a significant increase in the epidermal (suprabasal) thickness. In panel E, F of Figure 3, keratin filament clumping that interrupt the continuity of K1 staining can be detected. This phenomenon will be clearly visible in the captures of Figure 5 and Figure S1. The presence of peculiar substructures inside the dermal papillae is also interesting. These cannot be interpreted as the presence of sweat gland ducts because they are located inside the epidermis over the basal lamina and the K14 positive keratinocyte layer. They could resemble epidermal comedones.

**Figure 4:**
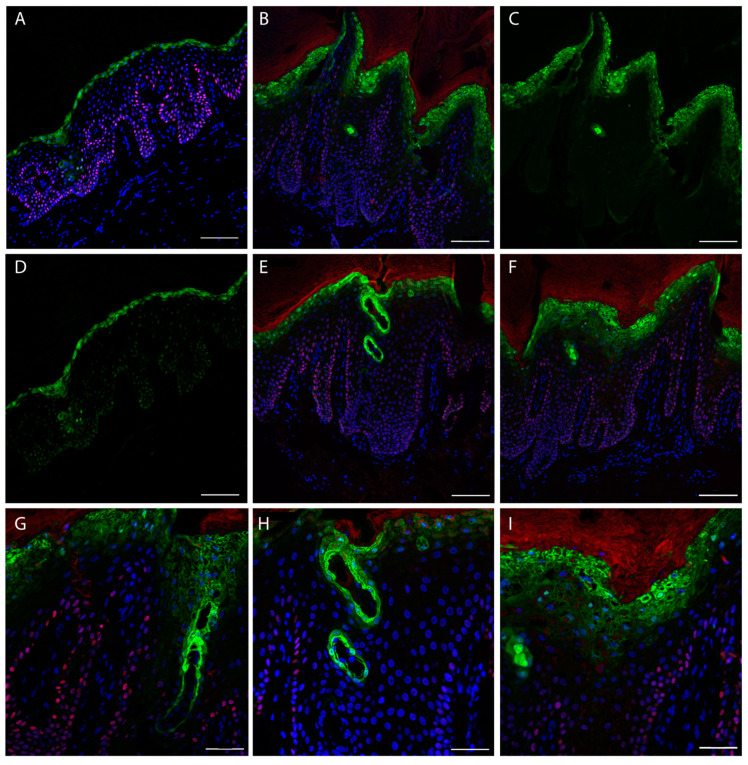
Skin confocal immunofluorescence analysis. Staining for p63 (red) and Loricrin (green). (**A**) Staining with p63 and loricrin merged with DAPI (Blue) of a control section. (**B**) Staining of a patient section. (**C**) Patient section (same of **B**) showing only loricrin signal. (**D**) Single channel acquisition of the control section showing only loricrin. (**E**,**F**) Patient skin stained with loricrin, p63 and DAPI. (**A**–**F)** Scale bar = 100 µm, 20× objective. (**G**–**I**), staining of patient sections with loricrin, p63 and DAPI at higher magnification; scale bar = 50 µm, 40× objective.

Since, a light K14 transgrediens expression (Figure 3I, Red) is visible in patient sections, we decided to also check the expression of p63 transcription factor (*TP63* gene), that is able to transcriptionally drive the *KRT14* gene, binding its promoter [27]. p63 is expressed in the nuclei of proliferating keratinocytes and it should be expressed in the basal layer [28,29]. The staining demonstrated mainly a basal layer localization in the control (Figure 4A). Conversely, the patient staining shows a degree of positivity of cells located in the granular layer (Figure 4B,E,F,G–I), and predominantly inside the dermal papillae. Notably, the staining with loricrin, that represent the late differentiation marker of the epidermis, is not restricted to the up granular layer as in the control (Figure 4A,D), but it shows a broadened expression in the patient skin granular layer (Figure 4B,C,E–I). Indeed, it is interesting that the peculiar structures identified by K1 staining resembling comedones, a result also evident with loricrin staining. This indicates that they contain differentiated corneocytes that should not be present inside the epidermis. Interestingly, these structures do not contain a basal layer, since is not present in any K14 staining (Figure 3C).

**Figure 5:**
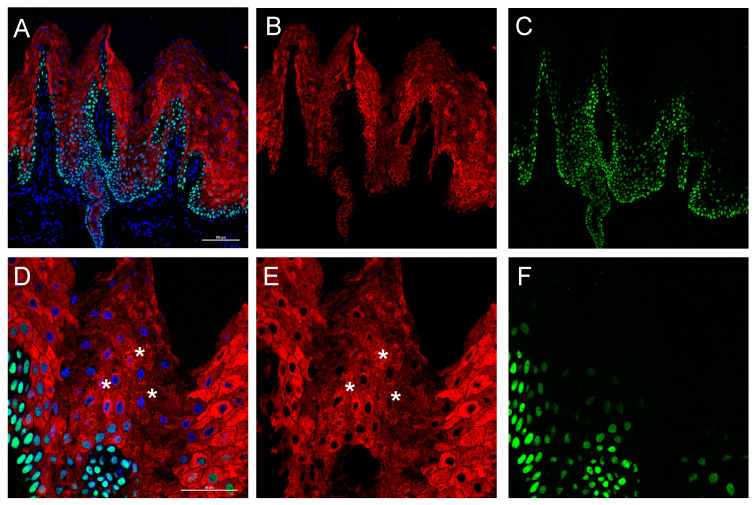
Skin confocal immunofluorescence analysis. Staining for p63 (Green) and K1 (Red). (**A**) p63 and K1 staining merged with DAPI (Blue) (**B**) K1 single channel. (**C**) p63 single channel (**A**–**C**, 20× objective). (**D**) High magnification capture (60× objective) showing nuclear localization of p63 and K1 filament clumping (stars, please see high magnification capture furnished in Figure S1); scale bars = 50 μm. (**E**) K1 single channel. (**F**) p63 single channel. Immunofluorescence analysis for p63 and K1 antigens. P63 is identified using Alexa fluor^®^488 igG (FITC green fluorescence), laser at 488 nm and 525/50 band pass filter while K1 with Alexa fluor^®^568 igG (TRITC red fluorescence), laser at 561 nm and 595/50 band pass filter. Nuclei detection was performed using DAPI (Blu fluorescence), laser at 404 nm and 450/50 band pass filter.

The p63 staining is not completely exhaustive about its localization and also shows interference by the corneum layer. We performed a further confocal analysis using Alexa fluor^®^488 (green) for p63 and Alexa fluor^®^568 (red) secondary antibody for K1 staining, this used a higher energy laser and a smaller pinhole. Moreover, a high magnification (60× objective) was used to better characterize the ultrastructural changes in the suprabasal epidermal layer of the patient. This epidermis, as previously evidenced, shows an increased thickness of the suprabasal, with a high number of nuclei positives to p63 staining (Figure 5A–C), not restricted to the basal layer. As it is appreciable in enlarged captures (Figure 5D–E, and Figure S1), p63 is expressed only in the nucleus of keratinocytes; in the K1 staining (Figure 5D,E, stars, and Figure S1), keratin clumping is visible. Moreover, software image analysis detected a higher number of p63 positive nuclei in the patient section (Figure S2).

### 2.3. Molecular Analysis

To analyze the molecular modification responsible of the palmoplantar keratoderma of our patient, DNA was extracted from peripheral blood lymphocytes (PBLs) and the sequence of keratins known to be associated to this phenotype was analyzed. From genomic DNA, we sequenced in a first instance *KRT9* and *KRT1* and, after, *KRT6* and *KR16*. This analysis did not show significant sequence variations, since we only detected a heterozygous variation in *KRT1* recognized as a previous described polymorphism in K1 (rs14024, discussed in patient presentation section), as well as another heterozygous variation in *KRT6*a also known as a polymorphism (rs17845411). Before taking into consideration other probable causes, we proceeded to extract the RNA from a skin biopsy of the hyperkeratotic area of the left palm of the patient and amplified the *KRT1* and *KRT9* cDNAs. As a result, we did not obtain sequence variations from *KRT9* but the direct sequence of the of *KRT1* CDS showed, at the 5′ end, a scrambling of the sequence (overlap of two sequences).

**Figure 6:**
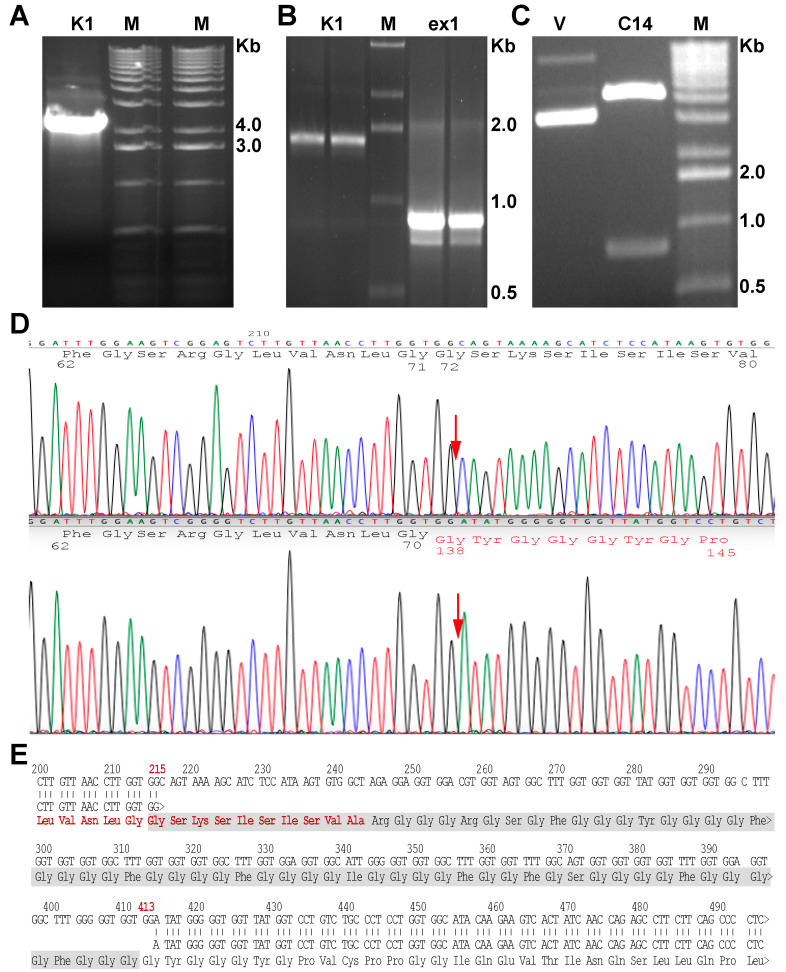
Molecular analysis. (**A**) Electrophoretic analysis of amplified *KRT1* from genomic DNA using to sequence the gene from PBLs DNA extract. (**B**) Electrophoresis of full length *KRT1* cDNA and fragment of exon 1 (right lanes), obtained from the reverse-transcription and amplification using RNA extracted from palmar skin biopsy. Right lanes show specific amplification of exon 1, which are evident by the smaller band due to the presence of the deletion. (**C**) Electrophoresis of a positive clone containing the deleted K1 exon 1, the vector was digested with BstXI restriction enzyme. (**D**) Electropherograms showing the sequence of the WT and mutated K1 of the patient obtained from cloned exon1 fragments. (**E**) Sequence alignment of WT and mutated K1, showing the deleted amino acids. The last 15 residues that belong to the ISIS box (total of 21 residues) are in red.

The electrophoretic analysis of the genomic *KRT1* amplification did not show any additional band (Figure 6A). To further investigate the source of the sequence scrambling, we performed a more accurate gel electrophoretic analysis running the full-length cDNA and a smaller fragment spanning only the first 850 base pairs. As is visible in Figure 6B, an additional band of low molecular weight is clearly visible in the short amplification (Figure 6B, right lane). From the electrophoretic analysis, it is appreciable that the shorter fragment is present in lower concentration (Figure 6B, right lane). To have a clear sequence reading, we cloned both fragments in PCR-2.1 cloning vector. Figure 6C shows the electrophoresis of the vector after the digestion with BstXI restriction enzyme, showing the presence of the deleted mutated insert. A restriction site for this enzyme is present inside the WT sequence insert, leading to the production of smaller fragments (see S2 image for details). We sequenced the clones and the upper fragment showed a normal K1 sequence (Figure 6D, upper panel), whereas the sequence analysis of the lower band detected the sequence deletion NM_006121.4:r.274_472del for a total of 198 nucleotides (215_413 considering from the ATG), corresponding to 67 amino acids (Figure 5D, lower panel). The exact point of the deletion is indicated by the red arrow (Figure 6D). This nucleotide modification give rise to the protein in frame deletion NP_006112.3:p.Gly71_Gly137del. The analysis of clones containing the *KRT1* 850bp fragment, those containing the shorter fragment represent approximatively the 15% of total clones, typical of a postzygotic mutation. This mutation is located in the head domain of K1, deleting a region of the V1 domain that involve the ISIS box functional domain [7]. This particular subdomain is composed of 22 amino acids (GGFGSRSLVNLGGSKSISISVA in K1), spanning from codon 60 to codon 82. Specifically, the mutation deletes the last 10 amino acids (Figure 4E,-GSKSISISVA).

### 2.4. Computational Model of Mutated K1 Interaction and Structure

**Figure 7:**
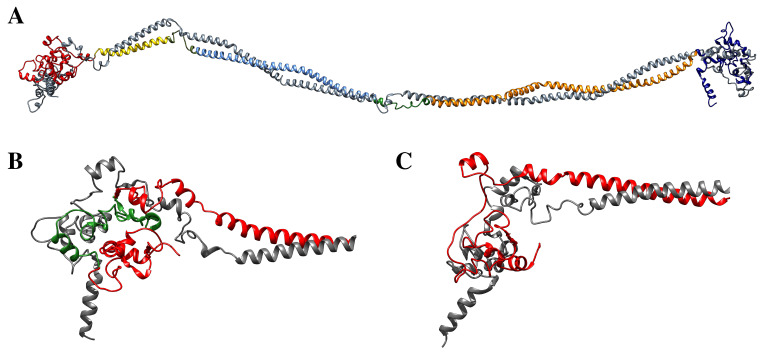
(**A**) Model of the K1/K10 dimer [16]. The major domains and the linker regions are identified by different colors. (red, head domain; yellow, coil 1A; cyan, coil 1B; orange, coil 2; blue, tail domain). K10 keratin is represented by gray ribbon and wires. (**B**) Detail of the head domain of the WT K1 keratin (red). The green color identifies the V1 domain. (**C**) Detail of the ΔK1/K10 dimer model, characterized by the absence of the V1 domain, used as a starting structure for molecular dynamics simulations.

To verify the impact of the K1 NP_006112.3:p.Gly71_Gly137del, a molecular model of the ΔK1/K10 dimer was generated [30], starting from the WT K1/K10 dimer model published by the Notman group [16] (Figure 7A) and using the NCBI reference sequence NP_006112.3 (Genbank ID NM_006121.4) for KRT1 mutation, in which the mutation was inserted. In particular, the NP_006112.3:p.Gly71_Gly137del corresponds to the loss of the V1 domain that is located in the “head” of the K1 structure (Figure 7B, red). However, due to the elastic nature of the keratin filaments, the molecular model of the ΔK1/K10 dimer suggests that K1 can still interact with K10, also after the deletion event (Figure 7C). 

To validate this hypothesis, two 30 ns long classical MD simulations were performed to gain an insight on the effects produced by the deletion on the structural arrangement and dynamical properties of the K1/K10 dimer.

**Figure 8:**
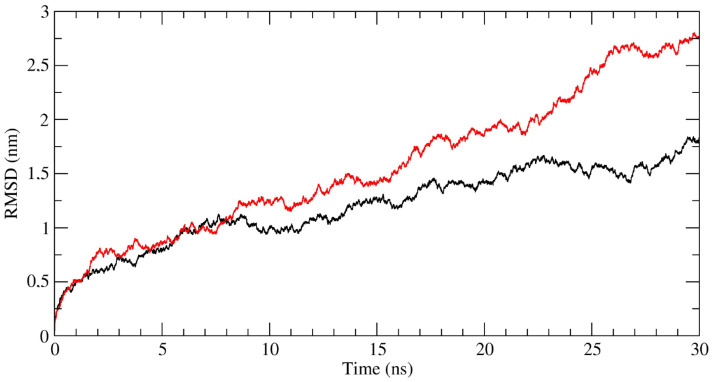
Time-dependent RMSD calculated for the WT (black line) and ΔK1/K10 (red line) dimers.

Analysis of MD trajectories indicates that the NP_006112.3:p.Gly71_Gly137del mutation largely alters the dynamics of the K1/K10 dimer. This result is clearly indicated by the RMSD analysis, describing the evolution of the sampled conformations in terms of distance from the starting structure [31,32,33]. Figure 8 shows the RMSD values calculated for the WT (black lines) and ΔK1/K10 (red line) dimers, and although far from converging, this trend suggests a large deviation occurring for the ΔK1/K10 complex which should lead to an almost impaired interaction. 

**Figure 9:**
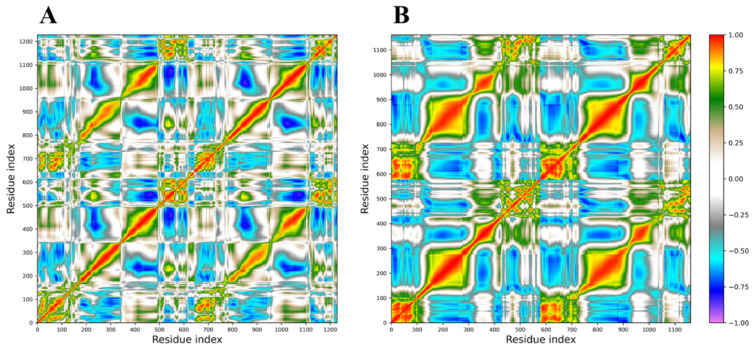
Dynamic cross-correlation matrices obtained from PCA analysis of the (**A**) WT (black line) and (**B**) ΔK1/K10 (red line) dimers trajectories. Positive correlations are identified by colors ranging from light brown to red, while negative correlations are described by colors ranging from grey to violet.

A PCA analysis of the trajectories was also performed, and the correlation matrices calculated from the covariance matrices are reported in Figure 9 for both the WT (A) and ΔK1/K10 (B) dimers. In these graphs, positive correlations*,* indicating a correlated motion between two residues, are identified by colors ranging from light brown to red, while negative correlations, indicating an anti-correlated motion, are described by colors ranging from grey to violet. The loss of the V1 region generates consistent effects on the internal motions of the protein, at the level of the complex but also for the isolated molecules, as indicated by the increase in both correlated and anti-correlated motions along the entire protein structures. These altered motions modify the overall flexibility of the protein complex, as confirmed by the calculation of the trace of the covariance matrices, corresponding to the sum of the atomic fluctuations. In fact, this parameter reaches the value of 1054.4 nm^2^ for the ΔK1/K10 dimer, while assumes the value of 727.8 nm^2^ for the WT dimer, confirming that the loss of V1 domain increases the structure flexibility, affecting the dynamic properties and consequently the activity of the dimer, explaining its unusual behavior. 

Finally, the interaction energies between the WT K1/K10 and the ΔK1/K10 keratin dimers were evaluated through the MM/GBSA method [34] to estimate how much the interaction energy between the two keratin filaments was reduced in the absence of the V1 domain. The MM/GBSA analysis identified a significative decrease in the interaction energy of about 135 kcal/mol in the ΔK1/K10 dimer, which is mainly attributed to the reduction of the hydrophobic contribution identified by the van der Waals energies (Table 1).

Even if this reduction in the binding energy is not sufficient to prevent the association of the two keratins, the results suggest an abnormal interaction between these keratin filaments. Moreover, we also tested in silico the possible pathogenicity of the mutation using Mutation Taster software [35]. The analysis was performed using the Ensembl transcript ID ENST00000252244 (Genbank ID NM_006121.4) and inserting the deletion found in the *KRT1* CDS of our patient. As a result, we obtained the prediction of “disease causing-long InDel” indicating that the mutation has high probability to be deleterious (Complete analysis in S3 file). This deletion mutation is not reported in the ClinVar database, but the variation VCV000015910/ NP_006112.3:p.Lys74Ile [7] falls inside the deleted sequence and is associated to a type of palmoplantar keratoderma.

## 3. Discussion

In this paper we presented a patient affected by a particular form of palmoplantar keratoderma. Interestingly, the patient represents a mosaic of this disease since the skin manifestations are localized only in the right palm. The molecular analysis showed that the disease can be classified as a mosaic form of epidermolytic hyperkeratosis, since a mutation in the *KRT1* gene was found in lesional skin, but not in DNA extracted from PBLs, thus indicating a postzygotic occurrence of the mutation. The dermatological symptoms do not involve other skin districts, only part of right palmar epidermis. However, we do not have data available about the possible presence of the mutation also in the germinal line of the patient. The literature [36] shows that is possible that the genetic transmission of a mutation from a mosaic parent to the offspring, if the mutation is present also in germline. If this happens, the progeny could be affected by a generalized disease, in this case by epidermolytic hyperkeratosis. Clinically, due to the characteristics of the palmar skin of the patient, we can define this entity as an epidermolytic palmar nevus. Up to now, nevoid presentations due to somatic mutations in *KRT1* have already been described [37], as well as in the companion *KRT10* [38]. Cases of mosaic epidermolytic ichthyosis have been described [39], others have not been genetically characterized [40]. A similar nevoid presentation was previously reported also for a deletion mutation in *KRT16* [21]. epidermal substructures resembling comedones present in our patient are also of particular interest. Scientific literature reports publications in which epidermolytic naevi associated with comedones are discussed [41], and this was also reported in a patient carrying a *KRT10* mutation [38]. Moreover, patients affected by comedonic nevi associated with epidermolytic hyperkeratosis have been reported, with histopathological findings compatible with a kind of keratin disease [40,42]. These observations support the hypothesis that the dysregulation of cell polarity due to KIF malformation can lead to the formation of comedone-like epidermal substructures. The transgrediens expression of K14 and of p63 could be correlated with the hyperproliferation of keratinocytes in the spinous and granular layers, leading to the increased epidermal thickness in patients’ palmar epidermis [8].

The reason of the particularity of this phenotype could reside also in the peculiar mutation found in the patients. We showed that this new deletion involves part of the head domain of K1, deleting the last 10 amino acids (Figure 4E) of the ISIS box subdomain. Notably, the mutation ablates the lysine 74 (numbered K73 in [7]), recognized as important for the crosslinking with other cytoskeleton proteins operated by transglutaminase 1 (TGase-1) enzyme [7,22,43]. This leads to a weakening of the keratin filament network of the involved epidermal layers [22].

Moreover, results obtained through carrying out an atomistic computational analysis integrates this experimentally observed scenario. The V1 domain of K1 was postulated to contribute for a large number of hydrophobic interactions associated to the interaction between K1/K10 head domains, thus leading to a significant contribution to the initial stability of the keratin dimer and KIF formation [16]. Interaction between the paired 1A and 2B domains of K1/10 and their V1 domains give rise to a complex structure responsible for the main physiological characteristics of IF filaments [16]. The large deletion found in the patients could destabilize the structure of the wildtype K1/K10 dimer. The calculated reduction in the binding energy, even though it is not sufficient to completely prevent the association between the two keratins filaments, leads to an anomalous assembly that may represent the atomistic cause underlying the observed altered function. Further, in silico investigation by the use of Mutation Taster software [35] also confirmed the possible deleterious role of the mutation.

Once again, we underline the role of V1 head domain of K1 protein in the organization of keratin filaments required for cytoskeleton stability of keratinocytes.

Moreover, the susceptibility of postzygotic mutation in keratin genes leading to peculiar mosaic or nevoid forms of skin diseases [6,15,21,37,38] could lead to the definition of the keratin epidermal nevoid disease.

## 4. Materials and Methods

### 4.1. Genetic Analysis

Total RNA extraction from biopsy of the left palmar area was performed using an RNeasy minikit (Qiagen, Crawley, UK). Reverse transcription was performed through Superscript II Reverse Transcriptase (Invitrogen, Waltham, MA, USA). Genomic DNA of the patient was extracted from peripheral blood using a Wizard^®^ Genomic DNA Purification kit (Promega, Hilden, Germany), according to manufacturer’s instructions. 

The following primers were used for keratin amplification: *KRT1* (GeneBank ID NM_006121.4) Forward 5′-AGTAAGGGAAGGAGCTAAACACTCC -3′ and K1 Reverse 5′-GTAGGTGGCAATCTCCAGATC-3′, K1 Reverse 5′-CATTCTCTGCATTTGTCCGCTTG-3′. Internal primers used for amplification and sequence: 5′-CAAGCGGACAAATGCAGAGAAT-3′, 5′-CCTTGGTCATATAAGCACCATCCACATC-3′, 5′-TCTATGGACAACAACCGCAGTCTC-3′, 5′-CCAACTTGCAGCAGTCCATCAGT-3′, 5′-GTAGGTGGCAATCTCCAGATC-3′.

For *KRT9* (GeneBank ID NM_000226.4) amplification and sequence, we used primers as in [43,44], for *KRT16* (GeneBank ID NM_005557.4) and *KRT6A* (GeneBank ID NM_005554.4) please see [19,21,45], for *KRT10 (*GeneBank ID *NM_000421.5*) please see [20]. For the reverse transcription and PCR amplification, total RNA (1 μg) was used for reverse transcription reaction with a SensiFAST^TM^ cDNA Synthesis kit (ThermoFisher scientific, Waltham, MA, USA). Amplification was performed by phusion hi fidelity taqman (ThermoFisher, Waltham, MA, USA).

PCR products were purified with QIAquick^®^ Gel Extraction kit (Qiagen, Hilden, Germany) and Sanger sequenced (3730 DNA Analyzer-Thermo Fisher Scientific, Waltham, MA, USA) using primers described above. Clone sequencing was performed using M13forward and M13reverse standard primers. ABI files were analyzed using MacVector^®^ (Version 17.5.4). Cloning was performed using pcr2.1 vector (TA Cloning ^TM^ kit, Thermofisher, Waltham, MA, USA) and DH5α competent bacteria according to standard protocols. Digestion Analysis BstXI (New England Biolabs, Ipswich, MA, USA, #R0113S), was performed using 1 μg of vector DNA, in a total volume of 50 μL using 0.5 μL of enzyme in 1× NEBuffer™ r3.1, incubated at 37 °C for 1 h.

### 4.2. Light Microscopy

Patient and control skin biopsies were analyzed with light microscopy, through hematoxylin-eosin staining according to standard methods.

### 4.3. Confocal Immunofluorescence Analysis

Skin sections were fixed in formalin 4%, permeabilized with Triton X-100 0.1% in PBS 1X and embedded in paraffin. Sections were incubated in heater for 1 h, washed in Bio-Clear (Bio-Optica) to remove paraffin and rehydrated with a decreasing alcohol concentration washes (100%-95%-80%-70%-50%-H_2_O, Sigma Aldrich, Gillingham, UK). After boiling in sodium citrate (0.01 M, pH 6) for antigen unmasking, sections were stored in sodium tetrahydroborate (*NaBH4*, Sigma Aldrich, St. Louis, MO, USA) at 4°C, overnight. After the incubated in blocking buffer (PBS1X + 5% goat serum) for 2 h, at room temperature, the following primary antibodies were: mouse polyclonal anti-K14 (LL02, Abcam, dilution 1:1000), rabbit polyclonal anti-K1 (Covance, dilution 1:1000) mouse polyclonal anti-p63 (Abcam Ab735, dilution 1:500), rabbit polyclonal anti-Loricrin (Covance, Dilution 1:1000). The following secondary antibodies: Alexa fluor^®^488 goat anti-rabbit igG (H + L) (Invitrogen, Carlsbad, CA, USA, dilution 1:1000) and Alexa fluor^®^568 goat anti-mouse igG (H + L) (Invitrogen, Carlsbad, CA, USA, dilution 1:1000). Nuclei staining was performed by DAPI (Thermo Fisher Scientific, Waltham, MA, USA, 5 mg/mL stock solution, used at dilution 1:1000). All antibodies were prepared in blocking buffer. Sections were covered by Prolong Antifade reagent (Invitrogen, USA). Images of each section were obtained using a confocal laser microscope NikonEclipse Ti. Laser at 405 nm was used for DAPI detection, a laser of 561 nm for the detection of Alexa fluor^®^568 and 488 nm for Alexa fluor^®^488. Signal analysis was performed using NIS Element AR4.00.04 (Nikon) software.

### 4.4. Computational Methods

#### 4.4.1. Molecular Models of Wild-Type and V1-Deleted K1/K10 Dimers

In the absence of available experimental structures, we used the molecular model of the K1/K10 keratin dimer published by the Notman group [16] as a reference structure (Figure 6A). The pDel71_138, corresponding to the V1-deleted K1/K10 model (from here named ΔK1/K10 dimer), was generated by removing the missing residues through the program UCSF Chimera [30]. The structure was reconnected using the PyMol sculpting module [46], which, acting similar to a real-time energy minimizer, allows for the remodeling of the peptide chain without introducing undesirable clashes or distortions. (For K1Genbank ID NM_006121.4, and NM_000421.3 for K10)

#### 4.4.2. Molecular Dynamics Simulations

The WT K1/K10 and the ΔK1/K10 dimers topology and coordinate files were generated using the Amber 16.0 [33] tLeap tool, parameterizing the proteins with the Amber ff19SB force field [31]. The systems were solvated with TIP3P water in a rectangular box with 14.0 Å between the protein surface and the box boundaries, and electrostatically neutralized by the addition of appropriate number of Na^+^ counterions. The systems were previously energy minimized for 2000 steps of steepest descent algorithm followed by 500 steps of conjugate gradient algorithm to eliminate close van der Waals contacts generated by the modeling procedure and then gradually heated from 0 to 300 °K in 1.0 ns, followed by constant pressure equilibration at 300 °K for 1.0 ns. Following this phase, 30 ns production of molecular dynamics (MD) runs were carried out with periodic boundary conditions in the NPT ensemble, at a temperature of 300 °K using a Langevin thermostat [32] and a constant pressure of 1.0 atm with isotropic molecule-based scaling [45]. Bond lengths involving bonds to hydrogens were constrained using the SHAKE algorithm [47]. Long-range electrostatic forces were calculated using the particle-mesh Ewald (PME) method [48]. The GPU-enabled PMEMD module of AMBER 16.0 package was used to perform the MD simulations. 

#### 4.4.3. Trajectory Analyses

RMSD and PCA analyses were performed using the *rms* module and the *covar*, *anaeig* modules of the GROMACS 2020.3 package [49], respectively. The elements of the correlation analysis (*C_ij_*) were computed as:

$$C_{ij}=\frac{\langle \Delta r_{i}\cdot \Delta r_{j}\rangle }{\sqrt{\langle \Delta r_{i}^{2}\rangle }\cdot \sqrt{\langle \Delta r_{j}^{2}\rangle }}$$

where Δ*r_i_* is the displacement from the mean position of the *i*-th atom and the 〈  〉 represents the time average over the entire trajectory. Positive *C_ij_* values represent a correlated motion between residues *i* and *j* (i.e., the residues move in the same direction). Negative values of *C_ij_* represent an anti-correlated motion between residues *i* and *j* (i.e., they move in opposite directions). Correlation matrix plots were generated using a custom *in-house* code. Generalized Born and surface area continuum solvation (MM/GBSA) analyses [50] were performed over the last 10 ns of both simulations, using the MMPBSA.py.MPI program implemented in the AMBER16 software using two nodes of the ENEA HPC cluster CRESCO6 [51].

#### 4.4.4. Mutation Taster Is Silico Analysis

The analysis was performed using the sequence identified by Genbank ID NM_006121.4, and the software indicated the nucleotidic deletion.

## Figures and Tables

**Table 1:** Results of the MM/GBSA analyses performed for the WT and ΔK1/K10 dimers.

Dimer	VdW (kcal/mol)	Electrostatic (kcal/mol)	Nonpolar Solvation (kcal/mol)	Polar Solvation (kcal/mol)	Binding Energy (kcal/mol)
WT K1/K10	−1489.2	−450.7	1100.2	−203.4	−1043.2
ΔK1/K10	−1218.8	−313.9	1007.1	−180.8	−906.5

## Data Availability

Not Applicable.

## Supplementary Materials

The following are available online at https://www.mdpi.com/article/10.3390/ijms22136901/s1.
