# Supplementary material for: Post Zygotic, Somatic, Deletion in KERATIN 1 V1 Domain Generates Structural Alteration of the K1/K10 Dimer, Producing a Monolateral Palmar Epidermolytic Nevus
Source: Int J Mol Sci. 2021 Jun 27;22(13):6901. doi: 10.3390/ijms22136901 (PMC8269197; doi:10.3390/ijms22136901)
Supplement: Supplementary file 1 [file ijms-22-06901-s001.zip › Figure S1.pdf]

Figure S1

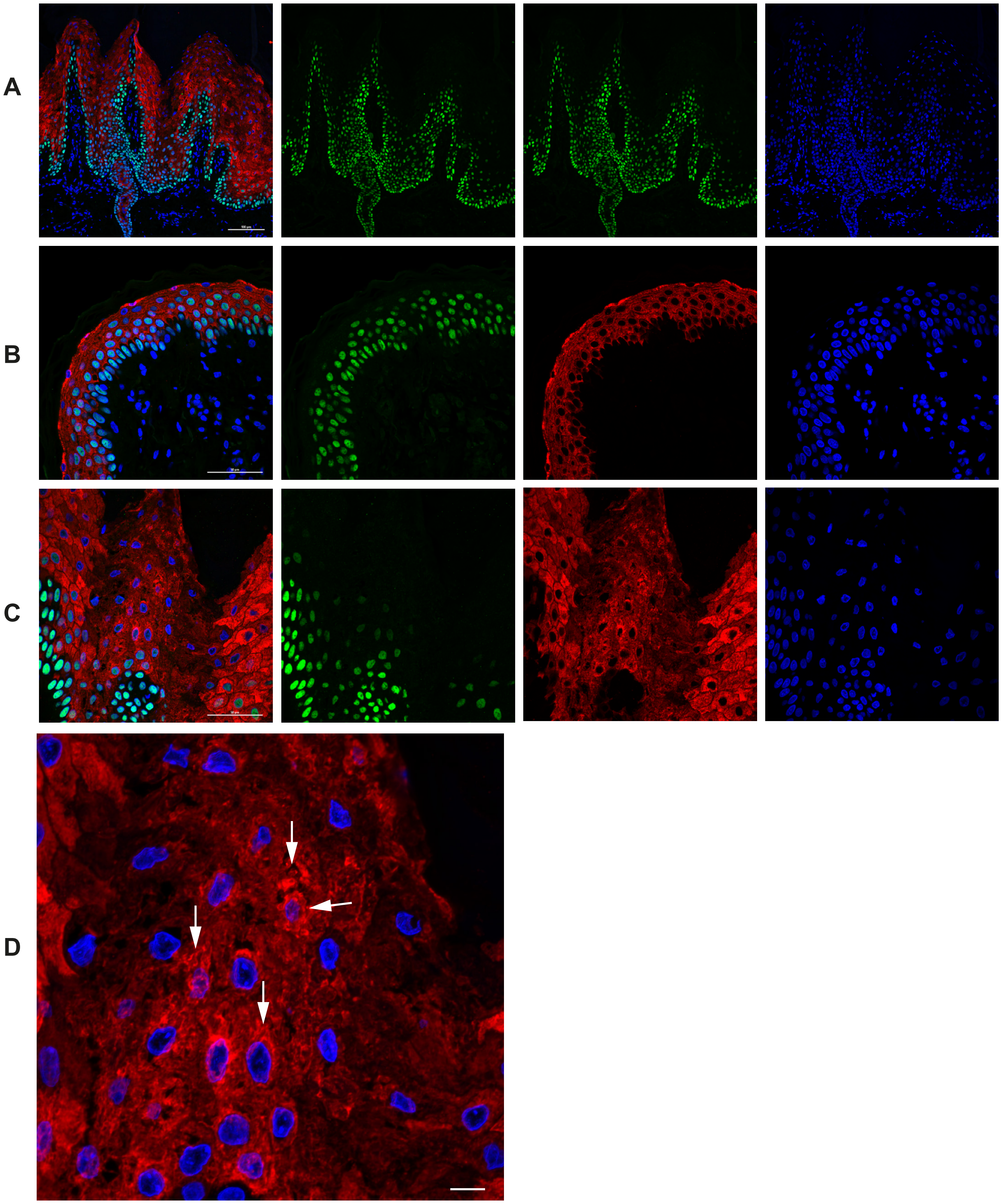

A, patient skin stained for p63 and K1 obtained with a 20x objective (merge, p63, K1, DAPI, bar=50μm). B, control skin stained for p63 and K1 obtained with a 60x objective. C, patient skin stained for p63 and K1 obtained with a 60x objective (merge, p63, K1, DAPI, bar=100μm).
D High magnification of patient skin stained for K1, the arrows indicate examples of keratin clumping. (merge, K1, DAPI, bar=10μm).
Immunofluorescence analysis for p63 and K1 antigens. P63 is identified using Alexa fluor®488 igG (FITC green fluorescence), laser at 488 nm and 525/50 band pass filter while K1 with Alexa fluor®568 igG (TRITC red fluorescence), laser at 561 nm and 595/50 band pass filter. Nuclei detection was performed using DAPI (Blu fluorescence), laser at 404 nm and 450/50 band pass filter.
