# Supplementary material for: Post Zygotic, Somatic, Deletion in KERATIN 1 V1 Domain Generates Structural Alteration of the K1/K10 Dimer, Producing a Monolateral Palmar Epidermolytic Nevus
Source: Int J Mol Sci. 2021 Jun 27;22(13):6901. doi: 10.3390/ijms22136901 (PMC8269197; doi:10.3390/ijms22136901)
Supplement: Supplementary file 1 [file ijms-22-06901-s001.zip › Figure S2.pdf]

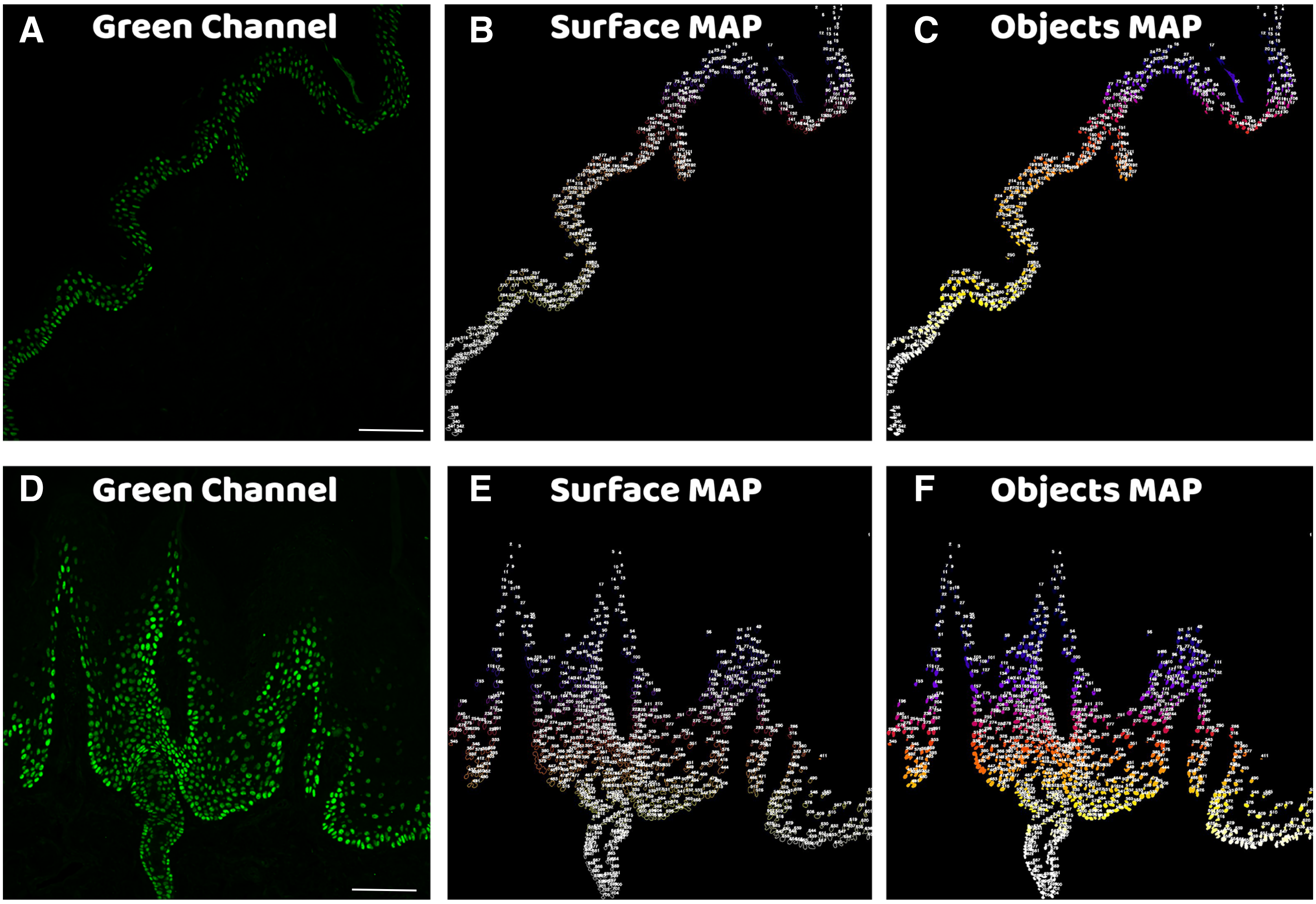

G

p63 positive nuclei and fluorescence quantification

Nuclei in 20x fields	Fluorescence Intensity Mean	Fluorescence Intensity SD
CTRL = 308,5 (Mean)+/-34,5	664710.8654	157097.8596
Patient = 682,5 (Mean)+/-22,5	1820911.449	453778.9407
Ratio Patient/Ctrl	2.739403767	

H

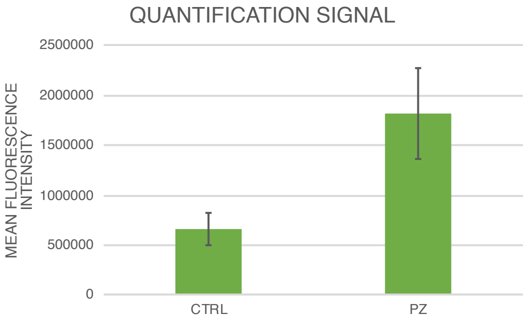

Figure S2.
A, confocal image of a representative control section (green channel, bar=100μm) B, confocal image of a representative patient section (green channel, same that in S1, bar=100μm). The count of p63 positive nuclei and the quantification of p63 signals obtained by immunofluorescence staining, were performed using ImageJ2 (Fijii, version 2.1.0/1.53c) software. Software required source Nikon ND2 format for the analysis.
We obtain the count of p63 nuclei, the surface maps (panels B, E for Ctrl and patient) and 3D objects (panels D, F for Ctrl and patient). G,H, statistics related to the analysis by analyze -> 3D-Objectes counter and fluorescence intensity.
Antibodies, lasers, Filters as described in S1.
