# Supplementary material for: Post Zygotic, Somatic, Deletion in KERATIN 1 V1 Domain Generates Structural Alteration of the K1/K10 Dimer, Producing a Monolateral Palmar Epidermolytic Nevus
Source: Int J Mol Sci. 2021 Jun 27;22(13):6901. doi: 10.3390/ijms22136901 (PMC8269197; doi:10.3390/ijms22136901)
Supplement: Supplementary file 1 [file ijms-22-06901-s001.zip › Supp S3 [MT]-Taster.pdf]

documentation

mutation t@sting

Alteration c.del215_413

Prediction disease causing - long InDel **Model: *complex_aae*, prob: 0.999999956067277** (explain)

Summary

- **amino acid sequence changed**
- **known disease mutation at this position (HGMD CM940998)**
- **protein features (might be) affected**
- **splice site changes**

hyperlink

analysed issue	analysis result
name of alteration	Garofalo
alteration (phys. location)	chr12:53073720_53073917delCCACCACCCCCAAAGCCACCTCCACCAAAACCACCACCACCACTGCCAAAACCACCAAAGCCACCACCCCCAATGCCACCTCCACCAAAGCCACCACCACCAAAGCCACCACCACCAAAGCCACCACCACCATAACCACCACCAAAGCCACTACCACGTCCACCTCCTCTAGCCACACTTATGGAGATGCTTTTACTG
HGNC symbol	KRT1
Ensembl transcript ID	ENST00000252244
Genbank transcript ID	NM_006121
UniProt peptide	P04264
alteration type	deletion
alteration region	CDS
DNA changes	c.216_413delCAGTAAAAGCATCTCCATAAGTGTGGCTAGAGGAGGTGGACGTGGTAGTGGCTTTGGTGGTGGTTATGGTGGTGGTGGCTTTGGTGGTGGTGGCTTTGGTGGTGGTGGCTTTGGTGGAGGTGGCATTGGGGGTGGTGGCTTTGGTGGTTTTGGCAGTGGTGGTGGTGGTTTTGGTGGAGGTGGCTTTGGGGGTGGTGG<br>cDNA.275_472delCAGTAAAAGCATCTCCATAAGTGTGGCTAGAGGAGGTGGACGTGGTAGTGGCTTTGGTGGTGGTTATGGTGGTGGTGGCTTTGGTGGTGGTGGCTTTGGTGGTGGTGGCTTTGGTGGAGGTGGCATTGGGGGTGGTGGCTTTGGTGGTTTTGGCAGTGGTGGTGGTGGTTTTGGTGGAGGTGGCTTTGGGGGTGGTGG<br>g.275_472delCAGTAAAAGCATCTCCATAAGTGTGGCTAGAGGAGGTGGACGTGGTAGTGGCTTTGGTGGTGGTTATGGTGGTGGTGGCTTTGGTGGTGGTGGCTTTGGTGGTGGTGGCTTTGGTGGAGGTGGCATTGGGGGTGGTGGCTTTGGTGGTTTTGGCAGTGGTGGTGGTGGTTTTGGTGGAGGTGGCTTTGGGGGTGGTGG
AA changes	deletion of more than 2 AA<br>G71- Score: -, G72- Score: -, S73- Score: -, K74- Score: -, S75- Score: -, I76- Score: -, S77- Score: -, I78- Score: -, S79- Score: -, V80- Score: -, A81- Score: -, R82- Score: -, G83- Score: -, G84- Score: -, G85- Score: -, R86- Score: -, G87- Score: -, S88- Score: -, G89- Score: -, F90- Score: -, G91- Score: -, G92- Score: -, G93- Score: -, Y94- Score: -, G95- Score: -, G96- Score: -, G97- Score: -, G98- Score: -, F99- Score: -, G100- Score: -, G101- Score: -, G102- Score: -, G103- Score: -, F104- Score: -, G105- Score: -, G106- Score: -, G107- Score: -, G108- Score: -, F109- Score: -, G110- Score: -, G111- Score: -, G112- Score: -, G113- Score: -, I114- Score: -, G115- Score: -, G116- Score: -, G117- Score: -, G118- Score: -, F119- Score: -, G120- Score: -, G121- Score: -, F122- Score: -, G123- Score: -, S124- Score: -, G125- Score: -, G126- Score: -, G127- Score: -, G128- Score: -, F129- Score: -, G130- Score: -, G131- Score: -, G132- Score: -, G133- Score: -, F134- Score: -, G135- Score: -, G136- Score: - explain score(s)
position(s) of altered AA<br>if AA alteration in CDS	71,72,73,74,75,76,77,78,79,80,81,82,83,84,85,86,87,88,89,90,91,92,93,94,95,96,97,98,99,100,101,102,103,104,105,106,107,108,109,110,111,112,113,114,115,116,117,118,119,120,121,122,123,124,125,126,127,128,129,130,131,132,133,134,135,136
frameshift	no
known variant	Variant was neither found in ExAC nor 1000G.<br>Search ExAC.<br><br>known disease mutation at this position, please check HGMD for details (HGMD ID CM940998)
regulatory features	H3K36me3, Histone, Histone 3 Lysine 36 Tri-Methylation<br>H3K27me3, Histone, Histone 3 Lysine 27 Tri-Methylation
phyloP / phastCons	

	PhyloP	PhastCons
(flanking)	-0.806	0
	1.762	0.097
	1.382	0.095
	-0.299	0.056
	1.884	0.087
	1.34	0.077
	-0.901	0.001
	0.656	0.005
	0.974	0.008
	-1.788	0
	1.495	0.56
	2.859	0.622
	0.059	0.523
	1.844	0.519
	0.452	0.336
	-0.093	0.244
	4.068	0.998
	3.056	0.992
	-1.156	0.003
	0.814	0.003
	0.45	0.002
	-2.746	0
	1.976	0.287
	0.71	0.294
	0.089	0.301
	5.43	1
	5.43	1
	-0.031	0.825
	1.072	0.825
	1.029	0.803
	-1.433	0.014
	2.443	0.976
	3.942	0.993
	-0.271	0.952
	1.811	0.975
	2.492	0.923
	-1.951	0.298
	1.821	0.497
	1.712	0.503
	0.284	0.425
	4.947	0.983
	3.361	0.981
	-0.049	0.464
	0.543	0.435
	0.515	0.062
	-1.294	0.04
	3.35	0.486
	0.875	0.465
	-0.017	0.426
	1.401	0.52
	0.526	0.481
	0.097	0.172
	1.9	0.175
	0.188	0.082
	-0.707	0.075
	2.348	0.903
	1.576	0.92
	0.246	0.854
	2.35	0.866
	0.905	0.797
	-1.57	0.247
	3.746	0.983
	2.298	0.989
	-0.263	0.977
	3.792	0.995
	3.792	0.989
	-0.929	0.116
	2.401	0.212
	0.481	0.09
	-2.02	0.001
	1.911	0.8
	1.233	0.719
	-0.387	0.269
	1.239	0.281
	0.26	0.024
	-0.96	0.018

2.382	0.981
5.04	1
1.163	1
2.789	1
1.957	0.959
-2.568	0.008
2.011	0.081
0.648	0.078
-2.768	0
2.124	0.972
5.713	0.981
-0.18	0.015
0.033	0.008
1.538	0.021
-1.487	0.001
3.48	0.944
0.417	0.498
0.021	0.346
1.925	0.34
-0.456	0.09
0.068	0.11
5.367	0.431
0.67	0.215
-0.733	0.056
1.454	0.089
0.663	0.088
0.436	0.088
0.272	0.092
1.714	0.115
0.136	0.023
1.428	0.027
0.34	0.019
-0.027	0.019
1.47	0.982
2.661	0.981
-0.553	0.789
1.502	0.916
3.082	0.928
-0.794	0.742
2.991	0.99
1.566	0.99
0.634	0.979
0.377	0.969
0.713	0.967
0.138	0.962
2.32	0.968
0.444	0.937
0.153	0.916
1.679	0.916
1.779	0.748
-0.84	0.016
0.565	0.018
1.626	0.021
-4.166	0
2.13	0.969
2.183	0.975
0.357	0.946
-0.977	0.023
1.912	0.074
-0.471	0.07
3.839	0.949
2.187	0.946
-0.147	0.754
0.336	0.778
3.949	0.936
-0.635	0.313
5.731	0.952
1.117	0.881
-0.088	0.002
-0.454	0
0.504	0
-1.855	0
5.657	0.92
1.618	0.886
0.338	0.471
1.015	0.361
-1.194	0.021
0.469	0.076
0.245	0.069
0.245	0.062
-0.42	0
-0.075	0
-1.331	0
-1.88	0
3.069	0.962
2.359	0.966
0.232	0.897
2.208	0.985
1.4	0.978
-0.468	0.926
3.87	1
3.414	1
1.136	0.994
1.999	0.967
-0.072	0.29
-0.003	0.271
0.643	0.301
1.893	0.324
0.353	0.24
2.043	0.292
1.607	0.276
0.068	0.051
0.417	0.021
0.343	0.007
-2.808	0
-0.174	0.001
0.162	0.015
0.041	0.081
2.1	0.925
2.404	0.953
0.779	0.939
0.917	0.918
1.072	0.859
-0.246	0.62
2.304	0.641

	1.153	0.453
	-0.208	0.009
	-0.115	0.001
	-0.063	0.001
	-0.037	0
	0.335	0
	-0.194	0
	0.016	0.003
(flanking)	1.455	0.948

explain score(s) and/or inspect your position(s) in in UCSC Genome Browser

splice sites

effect	gDNA position	score	wt detection sequence	exon-intron border
Acc gained	472	0.47	mu: GAGTCTTGTTAACCTTGGTGGATATGGGGGTGGTTATGGTC	|

distance from splice site 179

Kozak consensus sequence altered? N/A

conservation protein level for non-synonymous changes

species	match	gene	aa alignment
Human			71 GGFGSRSLVNLGGSKSISISVA RGGGRG SGFGGG YG GGGFGGGGFGGGGFGGGG IGGGGFGGFGSGGGGFGGGGFGGGGYGGGYGPVCP
mutated	partly conserved		30 --GSAGIINYQ-RRTTSSSTR RSGG-- ------ -- ---------GGGRFSSCG GGGGSFGA----GGGFGSRSLVNLGGYGGGYGPV
Ptroglodytes	no homologue		
Mmulatta	partly conserved	ENSMMUG00000016962	72 GGFGSRSLVNLGGSKSISISVA RGGGR- SGFGGG YG GGGFGGGGFGGGGFGGGGFGGGG IGGGGFGGFGSGGGGFGGGGFGGGYGGGYGPVC
Fcatus	partly conserved	ENSFCAG00000000291	72 GGFGSRSLVNLGGSKSISISVAGGGRGGFGG GGFGGG FGSGGGFGGGGFGGSGFGGGG FGGGGFGGGGFGGGGFGGSGFGGGGFGGGSFGPV
Mmusculus	partly conserved	ENSMUSG00000046834	72 SGFGSKSLMNLGGGRSISKSVA GGGGSFCGGFGGGSYG GGGFGGGSYGGGGFGGGS FGGGGFGGSGFGGGLGGGGGFGSGGGFGGGRFGS
Ggallus	no homologue		
Trubripes	no homologue		
Drerio	no homologue		
Dmelanogaster	no homologue		
Celegans	no homologue		
Xtropicalis	no homologue		

protein features

start (aa)	end (aa)	feature	details	
2	151	COMPBIAS	Gly/Phe/Ser-rich.	lost
2	179	REGION	Head.	lost
82	82	MOD_RES	Omega-N-methylarginine.	lost
180	215	REGION	Coil 1A.	might get lost (downstream of altered splice site)
180	489	REGION	Rod.	might get lost (downstream of altered splice site)
201	201	CONFLICT	L -> M (in Ref. 9; AAA36153).	might get lost (downstream of altered splice site)
206	206	CONFLICT	Q -> K (in Ref. 9; AAA36153).	might get lost (downstream of altered splice site)
216	234	REGION	Linker 1.	might get lost (downstream of altered splice site)
235	326	REGION	Coil 1B.	might get lost (downstream of altered splice site)
238	238	CONFLICT	L -> S (in Ref. 9; AAA36153).	might get lost (downstream of altered splice site)
276	276	MOD_RES	N6,N6-dimethyllysine.	might get lost (downstream of altered splice site)
295	295	MOD_RES	Phosphotyrosine.	might get lost (downstream of altered splice site)
297	297	MOD_RES	Phosphothreonine.	might get lost (downstream of altered splice site)
327	350	REGION	Linker 12.	might get lost (downstream of altered splice site)
344	344	MOD_RES	Phosphoserine (By similarity).	might get lost (downstream of altered splice site)
344	345	CONFLICT	SL -> QF (in Ref. 9; AAA36153).	might get lost (downstream of altered splice site)
351	489	REGION	Coil 2.	might get lost (downstream of altered splice site)
403	403	CONFLICT	R -> H (in Ref. 4; BAG36698).	might get lost (downstream of altered splice site)
404	404	CONFLICT	V -> M (in Ref. 9; AAA36153).	might get lost (downstream of altered splice site)
433	433	SITE	Stutter.	might get lost (downstream of altered splice site)
447	447	CONFLICT	L -> M (in Ref. 9; AAA36153).	might get lost (downstream of altered splice site)
463	463	CONFLICT	R -> C (in Ref. 9; AAA36153).	might get lost (downstream of altered splice site)
466	466	CONFLICT	Q -> H (in Ref. 9; AAA36153).	might get lost (downstream of altered splice site)
490	644	REGION	Tail.	might get lost (downstream of altered splice site)
502	641	COMPBIAS	Gly/Ser-rich.	might get lost (downstream of altered splice site)
504	504	CONFLICT	S -> T (in Ref. 9; AAA36153).	might get lost (downstream of altered splice site)
511	512	CONFLICT	TI -> SM (in Ref. 9; AAA36153).	might get lost (downstream of altered splice site)
564	564	CONFLICT	G -> S (in Ref. 9; AAA36153).	might get lost (downstream of altered splice site)
613	613	CONFLICT	I -> S (in Ref. 9; AAA36153).	might get lost (downstream of altered splice site)
638	638	CONFLICT	T -> S (in Ref. 9; AAA36153).	might get lost (downstream of altered splice site)

length of protein	deletion of more than 2 AA
AA sequence altered	yes
position of stopcodon in wt / mu CDS	1935 / 1737
position (AA) of stopcodon in wt / mu AA sequence	645 / 579
position of stopcodon in wt / mu cDNA	1994 / 1796
poly(A) signal	N/A
conservation nucleotide level for all changes - no scoring up to now	N/A
position of start ATG in wt / mu cDNA	60 / 60
chromosome	12
strand	-1
last intron/exon boundary	1570
theoretical NMD boundary in CDS	1460
length of CDS	1935
coding sequence (CDS) position	215 / 414
cDNA position (for ins/del: last normal base / first normal base)	274 / 473
gDNA position (for ins/del: last normal base / first normal base)	274 / 473
chromosomal position (for ins/del: last normal base / first normal base)	53073719 / 53073918

original gDNA sequence snippet
AGTCTTGTTAACCTTGGTGG**CAGTAAAAGCATCTCCATAAGTGTGGCTAGAGGAGGTGGACGTGGTAGTGGCTTTGGTGGTGGTTATGGTGGTGGTGGCTTTGGTGGTGGTGGCTTTGGTGGTGGTGGCTTTGGTGGAGGTGGCATTGGGGGTGGTGGCTTTGGTGGTTTTGGCAGTGGTGGTGGTGGTTTTGGTGGAGGTGGCTTTGGGGGTGGTGG**ATATGGGGGTGGTTATGGTC

altered gDNA sequence snippet
AGTCTTGTTAACCTTGGTGGATATGGGGGTGGTTATGGTC

original cDNA sequence snippet
AGTCTTGTTAACCTTGGTGG**CAGTAAAAGCATCTCCATAAGTGTGGCTAGAGGAGGTGGACGTGGTAGTGGCTTTGGTGGTGGTTATGGTGGTGGTGGCTTTGGTGGTGGTGGCTTTGGTGGTGGTGGCTTTGGTGGAGGTGGCATTGGGGGTGGTGGCTTTGGTGGTTTTGGCAGTGGTGGTGGTGGTTTTGGTGGAGGTGGCTTTGGGGGTGGTGG**ATATGGGGGTGGTTATGGTC

altered cDNA sequence snippet
AGTCTTGTTAACCTTGGTGGATATGGGGGTGGTTATGGTC

wildtype AA sequence
```
MSRQFSSRSG YRSGGGFSSG SAGIINYQRR TTSSSTRRSG GGGGRFSSCG GGGGSFGAGG
GFGSRSLVNL GGSKSISISV ARGGGRGSGF GGGYGGGGFG GGGFGGGGFG GGGIGGGGFG
GFGSGGGGFG GGGFGGGGYG GGYGPVCPPG GIQEVTINQS LLQPLNVEID PEIQKVKSRE
REQIKSLNNQ FASFIDKVRF LEQQNQVLQT KWELLQQVDT STRTHNLEPY FESFINNLRR
RVDQLKSDQS RLDSELKNMQ DMVEDYRNKY EDEINKRTNA ENEFVTIKKD VDGAYMTKVD
LQAKLDNLQQ EIDFLTALYQ AELSQMQTQI SETNVILSMD NNRSLDLDSI IAEVKAQYED
IAQKSKAEAE SLYQSKYEEL QITAGRHGDS VRNSKIEISE LNRVIQRLRS EIDNVKKQIS
NLQQSISDAE QRGENALKDA KNKLNDLEDA LQQAKEDLAR LLRDYQELMN TKLALDLEIA
TYRTLLEGEE SRMSGECAPN VSVSVSTSHT TISGGGSRGG GGGGYGSGGS SYGSGGGSYG
SGGGGGGGRG SYGSGGSSYG SGGGSYGSGG GGGGHGSYGS GSSSGGYRGG SGGGGGGSSG
GRGSGGGSSG GSIGGRGSSS GGVKSSGGSS SVKFVSTTYS GVTR*
```

mutated AA sequence
```
MSRQFSSRSG YRSGGGFSSG SAGIINYQRR TTSSSTRRSG GGGGRFSSCG GGGGSFGAGG
GFGSRSLVNL GGYGGGYGPV CPPGGIQEVT INQSLLQPLN VEIDPEIQKV KSREREQIKS
LNNQFASFID KVRFLEQQNQ VLQTKWELLQ QVDTSTRTHN LEPYFESFIN NLRRRVDQLK
SDQSRLDSEL KNMQDMVEDY RNKYEDEINK RTNAENEFVT IKKDVDGAYM TKVDLQAKLD
NLQQEIDFLT ALYQAELSQM QTQISETNVI LSMDNNRSLD LDSIIAEVKA QYEDIAQKSK
AEAESLYQSK YEELQITAGR HGDSVRNSKI EISELNRVIQ RLRSEIDNVK KQISNLQQSI
SDAEQRGENA LKDAKNKLND LEDALQQAKE DLARLLRDYQ ELMNTKLALD LEIATYRTLL
EGEESRMSGE CAPNVSVSVS TSHTTISGGG SRGGGGGGYG SGGSSYGSGG GSYGSGGGGG
GGRGSYGSGG SSYGSGGGSY GSGGGGGGHG SYGSGSSSGG YRGGSGGGGG GSSGGRGSGG
GSSGGSIGGR GSSSGGVKSS GGSSSVKFVS TTYSGVTR*
```

speed 0.13 s

Report bugs / help us to improve MutationTaster!

All positions are in basepairs (bp) if not explicitly stated differently.
AA/aa: amino acid; CDS: coding sequence; mu: mutated; NMD: nonsense-mediated mRNA decay; nt: nucleotide; wt: wildtype; TGP: 1000 Genomes Project
